# Elevated levels of Dickkopf-related protein 3 in seminal plasma of prostate cancer patients

**DOI:** 10.1186/1479-5876-9-193

**Published:** 2011-11-10

**Authors:** Christoph Zenzmaier, Martin Heitz, Helmut Klocker, Marion Buck, Robert A Gardiner, Peter Berger

**Affiliations:** 1 Institute for Biomedical Aging Research, Austrian Academy of Sciences, Innsbruck, Austria; 2 Department of Urology, Division of Experimental Urology, Innsbruck Medical University, Austria; 3 University of Queensland Centre for Clinical Research, Royal Brisbane Hospital, Australia

**Keywords:** Dickkopf-3, Dkk-3, IEMA, prostate-specific antigen, seminal fluid

## Abstract

**Background:**

Expression of Dkk-3, a secreted putative tumor suppressor, is altered in age-related proliferative disorders of the human prostate. We now investigated the suitability of Dkk-3 as a diagnostic biomarker for prostate cancer (PCa) in seminal plasma (SP).

**Methods:**

SP samples were obtained from 81 patients prior to TRUS-guided prostate biopsies on the basis of elevated serum prostate-specific antigen (PSA; > 4 ng/mL) levels and/or abnormal digital rectal examination. A sensitive indirect immunoenzymometric assay for Dkk-3 was developed and characterized in detail. SP Dkk-3 and PSA levels were determined and normalized to total SP protein. The diagnostic accuracies of single markers including serum PSA and multivariate models to discriminate patients with positive (N = 40) and negative (N = 41) biopsy findings were investigated.

**Results:**

Biopsy-confirmed PCa showed significantly higher SP Dkk-3 levels (100.9 ± 12.3 vs. 69.2 ± 9.4 fmol/mg; *p *= 0.026). Diagnostic accuracy (AUC) of SP Dkk-3 levels (0.633) was enhanced in multivariate models by including serum PSA (model A; AUC 0.658) or both, serum and SP PSA levels (model B; AUC 0.710). In a subpopulation with clinical follow-up > 3 years post-biopsy to ensure veracity of negative biopsy status (positive biopsy N = 21; negative biopsy N = 25) AUCs for SP Dkk-3, model A and B increased to 0.667, 0.724 and 0.777, respectively.

**Conclusions:**

In multivariate models to detect PCa, inclusion of SP Dkk-3 levels, which were significantly elevated in biopsy-confirmed PCa patients, improved the diagnostic performance compared with serum PSA only.

## Background

The secreted glycoprotein Dickkopf-related protein 3 (Dkk-3) is the most divergent member of the human Dickkopf family [1,2] that, in contrast to other family members, does not modulate Wnt signaling [3,4]. Dkk-3 has been proposed to represent a novel tumor suppressor since gene expression is downregulated in various tumor cells [5-9] and hypermethylation of its promoter correlates with cancer occurrence [10,11].

Our previous results indicated a possible link between Dkk-3 expression and age-associated processes in the human prostate. Serial analysis of gene expression (SAGE) of human prostate basal epithelial cells revealed specific induction of *DKK3 *gene during cellular senescence *in vitro *[12]. Moreover, Dkk-3 was found downregulated *in vivo *in prostate epithelium of patients suffering from the age-related prostatic conditions benign prostatic hyperplasia (BPH) and prostate cancer (PCa), with this downregulation counterbalanced by increased Dkk-3 protein expression in the endothelial cells of the tumor neovasculature [13].

This study was intended to assess changes in Dkk-3 content in prostatic fluid in these two conditions and its potential as a diagnostic marker for PCa. Since prostatic secretions constitute approximately 30% of seminal fluid volume [14], of all readily obtainable body fluids, ejaculate supernatant (seminal plasma; SP) reflects the contemporary status of the prostate best. We previously demonstrated that Dkk-3 could be detected in SP in apparently healthy donors at concentrations of 2.59 ± 0.41 nmol/L (range 1.62-5.25 nmol/L) that was higher that plasma Dkk-3 levels (1.22 ± 0.04 nmol/L) but much less than Dkk-3 levels found in cerebrospinal fluid (28.2 ± 1.3 nmol/L) [15]. SP Dkk-3 and SP prostate-specific antigen (PSA) levels were analyzed from 81 patients who underwent transrectal ultrasound (TRUS)-guided prostate biopsies due to elevated serum PSA levels and/or abnormal digital rectal examination (DRE). Ejaculate specimens were obtained prior to biopsy. SP Dkk-3 and SP PSA levels were compared, alone and in combination with serum PSA, to the diagnostic performance of serum PSA alone to detect PCa in univariate and multivariate models.

## Methods

### Subjects - SP samples

Eighty-one patients who were to undergo TRUS-guided biopsies because they had an abnormal serum PSA level (> 4 ng/mL) and/or abnormal DRE were recruited into the study from the outpatient department or from private rooms of urologists at Royal Brisbane and Women's Hospital (RBWH) between 1996 and 2009. Of this group, 40 men were confirmed to have cancer and 41 men had negative biopsies. Subsequently, patients with negative TRUS biopsy findings were followed with PSA and DREs and with further TRUS biopsies as indicated in selected cases to ensure the veracity of the TRUS-negative findings. A subgroup of 25 patients with negative and 21 with positive biopsy had a clinical follow-up of > 3 years (collected between 1996 and 08/2007). This subset of patients had been selected from our archive because we were as sure as we can be that those designated not to have prostate cancer were true negatives. All SP specimens were obtained before biopsy and treatment and after giving informed consent. Patients collected ejaculate via masturbation into urine collection jars containing 20 mL of Hanks balanced salt solution in the morning at home and brought them to the hospital within 2 hours of collection (without freezing or cooling, i.e. at temperatures from ~5-25°C) where they were processed within 15 minutes after arrival. Samples were layered over 10 mL of 64% isotonic Percoll (Amersham Biosciences) and centrifuged at 2200 rpm for 30 min at 4°C. The ejaculate supernatants (SP) were removed, aliquoted and stored at -70°C until further processing (as reported previously [16,17]). The study had the approval of the ethics committees of the RBWH and University of Queensland.

### Establishment of a sensitive Dkk-3 assay

**Figure 1:**
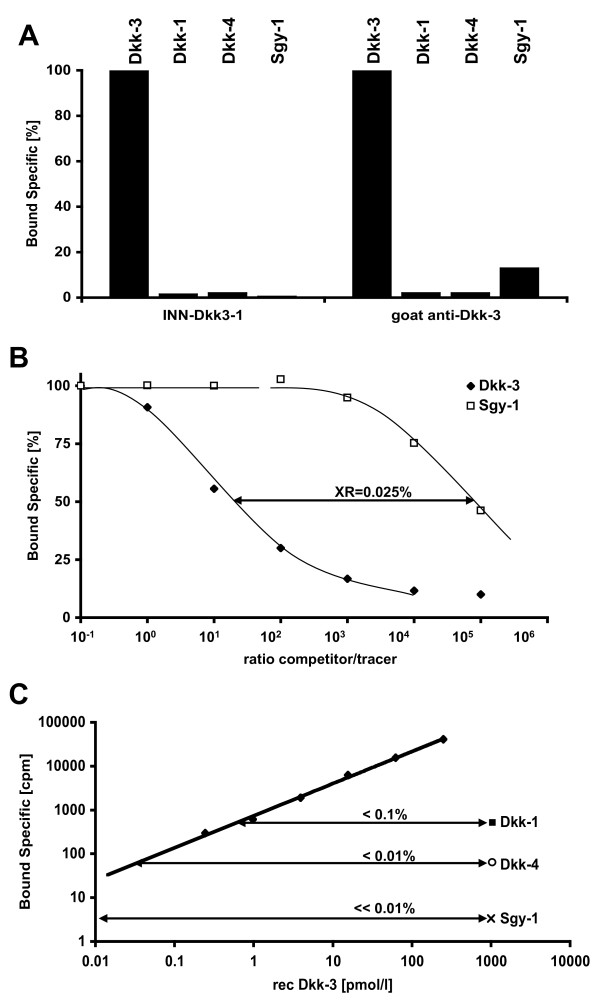
**Establishment of the Dkk-3 assay**. mAb INN-Dkk3-1 and goat polyclonal antibody (R&D Systems) showed no significant cross-reactivities with homologous proteins Dkk-1 and Dkk-4. The polyclonal antibody (5 μg/mL) showed weak reactivity for the homologous protein Sgy-1 in a direct binding RIA (A). In a competitive RIA the goat polyclonal antibody showed a cross-reactivity (XR) of 0.025% for Sgy-1 (B). A standard curve from 0.25 pmol/L - 250 pmol/L Dkk-3 in comparison to the homologous proteins at a concentration of 1000 pmol/L revealed cross-reactivities in the sandwich assay of < 0.01% (C). The established sandwich assay had a recovery of 92% and sensitivity of 1 pmol/L. The intra- and inter-assay variances (%CV) at a protein concentration of 40 pmol/L were 11% and 13%, respectively.

A series of six monoclonal Antibodies (mAb) INN(sbruck)-Dkk3-1 to INN-Dkk3-6 as previously described [18] and an affinity purified polyclonal goat anti Dkk-3 antibody (R&D Systems Cat.#AF1118) were characterized by radioimmunoassays (RIA) according to affinity, molecular epitope localization and specificity with native (untagged) Dkk-3 and the commercially available homologous proteins Dkk-1, Dkk-4 and Sgy-1 (R&D Systems) as described elsewhere [18]. The antibodies with the highest affinity towards Dkk-3 (mAb INN-Dkk3-1 and the goat polyclonal antibody) showed no significant reactions towards the homologous proteins Dkk-1 and Dkk-4 however the polyclonal antibody when tested at a concentration of 5 μg/mL reacted slightly with the homologous protein Sgy-1 (Figure 1A). In competitive RIA to assess cross-reactivity increasing concentrations of Dkk-3 or Sgy-1 competed with ^125^I labeled Dkk-3 (approximately 25,000 cpm) for binding to 30 ng of polyclonal goat anti Dkk-3 antibody in 300 μL total reaction volume (1% BSA/PBS) overnight at 4°C. Separation of bound from free tracer was performed as described [18], and cross-reactivity towards Sgy-1 was determined to be 0.025% (Figure 1B). Epitope mapping by polypeptide screening revealed localization of the INN-Dkk3-1 epitope in the N-terminal region (aminoacids 21-147) while the polyclonal antibody predominantly reacted with the C-terminal region (aminoacids 200-350; data not shown).

A sensitive sandwich immunoradiometric assay (IRMA) was established with mAb INN-Dkk3-1 (coating antibody) and the ^125^l labeled goat polyclonal antibody (R&D Systems) as described previously [19]. All samples were run in duplicate and results calculated from specific mean signal (mean - zero standard). Native i.e. untagged Dkk-3 purified via HPLC (anion exchange followed by size exclusion chromatography) from conditioned media of stably transfected LNCaP cells [13] was used as standard. Cross-reactivity was shown to be < 0.1% for Sgy-1 and < 0.01% for Dkk-1 and Dkk-4 (Figure 1C).

To increase sensitivity and to allow higher sample throughput, the assay was commuted to a 96-well plate based indirect immunoenzymometric assay (IEMA) described previously [18] by replacement of the ^125^l labeled antibody by a biotinylated variant of the polyclonal goat anti Dkk-3 antibody (R&D Systems Cat.#BAF1118). All samples were run in duplicate and results were calculated from specific mean signal (mean - zero standard). Recovery was 92% and sensitivity 1 pmol/L. The intra- and inter-assay variances (%CV) at a protein concentration of 40 pmol/L were 11% and 13%, respectively.

### PSA and total protein measurements

Serum PSA was assayed using a fluoroimmunoenzymetric two-site assay (Medics 1200 Dx AIA, Tosoh Corp.). SP PSA values were analyzed by the ADVIA Centaur PSA assay (Siemens Healthcare Diagnostics) at a dilution of 1:5000. SP Dkk-3 and PSA levels were normalized to total protein levels determined by the Pierce BCA Protein Assay Kit (Thermo Scientific).

### Statistical analyses

Assay sensitivity was defined as the mean signal of non-specific binding (NSB = zero standard) plus three standard deviations (NSB + 3SD). Percent coefficient of variation (%CV) was used as a measure of dispersion about the mean and expressed SD as a percentage of the mean.

Statistical differences among groups were calculated by Mann-Whitney test. Multivariate logistic regression was used to analyze correlations between investigated markers and PCa diagnostic status and the quality of the obtained models were estimated by Hosmer-Lemeshow goodness-of-fit tests. The diagnostic performance of the tested variables and the multivariate models was evaluated by plotting the receiver-operating characteristics (ROC) curve and calculating the area under the ROC curve (AUC). Associations between the investigated markers, age at time of the ejaculation specimen and Gleason score were evaluated using Pearson correlation analysis or Spearman's rank correlation analysis when data belonged to categorial variables.

All statistical analyses were performed using PASW Statistics 18 (IBM).

## Results

The characteristics of all 81 patients and the 46 patients with a follow-up of > 3 years are summarized in Table 1. Serum PSA as well as SP Dkk-3 levels (characteristics of the sandwich assay depicted in Figure 1) were found significantly higher in the PCa confirmed patients in both, the all patients cohort as well as the > 3 year follow-up subgroup. SP PSA levels were although also slightly elevated not significantly different. A multivariate logistic regression model based on serum PSA and SP Dkk-3 levels (model A) significantly discriminated negative from positive biopsies in both cohorts (Table 2; all patients: *p *= 0.007, predictive accuracy = 64.2%; follow-up > 3 years: *p *= 0.007, predictive accuracy = 73.9). Although not significantly different between patients with positive and negative biopsies, inclusion of SP PSA (model B) increased the predictive accuracy of the model for the cohort of all patients.

**Table 1:** Characteristics of the study cohort.

all patients			
**Characteristic**	**Negative biopsy**	**Positive biopsy**	***p *value**

**Patients, N**	**41**	**40**	
Age*, yr			0.981
Mean ± SEM	61.0 ± 1.3	61.5 ± 1.3	
Median (IQR)	62.5 (57.1 - 67.0)	61.9 (55.6 - 66.6)	
serum PSA [ng/mL]			0.026
Mean ± SEM	7.5 ± 1.0	11.6 ± 1.9	
Median (IQR)	6.2 (4.1 - 8.9)	7.3 (5.8 - 13.3)	
SP Dkk-3 [fmol/mg]**			0.039
Mean ± SEM	69.2 ± 9.4	100.9 ± 12.3	
Median (IQR)	55.7 (35.8 - 91.2)	74.5 (41.7 - 124.5)	
SP PSA [μg/mg]**			0.317
Mean ± SEM	23.8 ± 3.3	28.0 ± 3.5	
Median (IQR)	16.8 (8.1 - 35.9)	19.9 (10.8 - 42.5)	
Biopsy Gleason score			
≤ 6		6	
7		26	
≥ 8		8	

clinical follow-up > 3 yr			

Characteristic	Negative biopsy	Positive biopsy	*p *value

Patients, N	25	21	
Age*, yr			0.700
Mean ± SEM	63.1 ± 1.6	61.6 ± 2.3	
Median (IQR)	64.0 (60.9 - 68.9)	60.5 (53.7 - 69.8)	
serum PSA [ng/mL]			0.012
Mean ± SEM	8.0 ± 1.6	14.3 ± 3.3	
Median (IQR)	6.2 (3.6 - 10.4)	9.2 (6.5 - 16.0)	
SP Dkk-3 [fmol/mg]**			0.027
Mean ± SEM	56.3 ± 8.4	100.0 ± 16.6	
Median (IQR)	49.6 (22.3 - 76.4)	76.3 (35.9 - 169.4)	
SP PSA [μg/mg]**			0.474
Mean ± SEM	23.4 ± 4.5	27.4 ± 5.1	
Median (IQR)	16.8 (7.3 - 40.1)	19.5 (12.7 - 35.0)	
Biopsy Gleason score			
≤ 6		3	
7		14	
≥ 8		4	

SEM, standard error of the mean; IQR, interquartile range; PSA, prostate-specific antigen; SP, seminal plasma; * at time of ejaculate specimen; ** per total protein; *p *values determined by Mann-Whitney test.

**Table 2:** Multivariate logistic regression analyses to asses the predictive value in prostate cancer detection.

Cohort	Variable	β-coefficient	OR	95% CI	*p*-value	Predictive accuracy %	H-L *p*-value
all patients	model A				0.007	64.2	0.979
	serum PSA	0.071	1.074	0.997 - 1.157	0.06		
	SP Dkk-3	0.008	1.008	1.000 - 1.016	0.039		
	model B				0.007	69.1	0.737
	serum PSA	0.07	1.072	0.996 - 1.002	0.063		
	SP Dkk-3	0.01	1.010	1.002 - 1.018	0.019		
	SP PSA	0.017	1.018	0.995 - 1.041	0.13		
clinical follow-up > 3 yr	model A				0.005	73.9	0.691
	serum PSA	0.071	1.074	0.992 - 1.164	0.08		
	SP Dkk-3	0.014	1.015	1.002 - 1.027	0.02		
	model B				0.007	73.9	0.806
	serum PSA	0.069	1.072	0.991 - 1.159	0.083		
	SP Dkk-3	0.016	1.016	1.004 - 1.029	0.012		
	SP PSA	0.018	1.018	0.988 - 1.049	0.238		

OR, odds ration; CI, confidence intervall; H-L, Hosmer-Lemeshow test; PSA, prostate-specific antigen; SP, seminal plasma.

**Figure 2:**
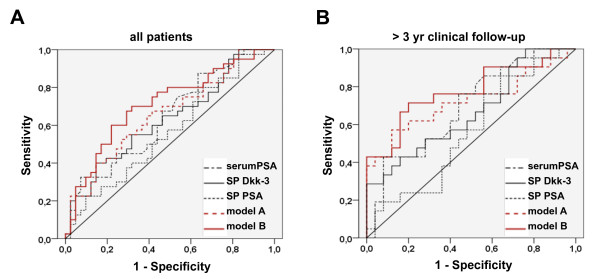
**Receiver operator characteristics (ROC) curves**. ROC curves of serum prostate-specific antigen (PSA), seminal plasma (SP) Dkk-3 and SP PSA levels and the multivariate models based on serum PSA and SP Dkk-3 (model A) or on all three individual markers (model B) for (A) the entire cohort and (B) patients with > 3 years clinical follow-up.

The diagnostic performances of the univariate test variables and the multivariate models were determined by ROC analyses (Figure 2 and Table 3). AUCs, as a measure for diagnostic accuracy, for serum PSA, SP Dkk-3, SP PSA, model A and model B were 0.644, 0.633, 0.565, 0.658 and 0.710 in the cohort of all patients and 0.696, 0.667, 0.562, 0.724 and 0.777 in patients with > 3 years clinical follow-up, respectively, showing that the multiplex models had higher AUCs in both cohorts compared with the single markers and model B higher accuracy compared with model A. Moreover, except for SP PSA all AUCs where higher in the > 3 year follow-up cohort.

**Table 3:** Determination of diagnostic accuracy.

	all patients		clinical follow-up > 3 yr	
		
	AUC	95% CI	AUC	95% CI
serum PSA	0.644	0.524 - 0.763	0.696	0.544 - 0.848
SP Dkk-3	0.633	0.512 - 0.754	0.667	0.509 - 0.825
SP PSA	0.565	0.439 - 0.690	0.562	0.394 - 0.730
model A	0.658	0.539 - 0.776	0.724	0.568 - 0.880
model B	0.710	0.597 - 0.824	0.777	0.638 - 0.917

Areas under the receiver operator characteristics curve for univariate test variables and multivariate models.

AUC, area under the curve; CI confidence intervall; PSA, prostate-specific antigen; SP, seminal plasma.

Lastly, correlations between the individual markers, age and biopsy Gleason score were analyzed (Table 4). SP Dkk-3 and SP PSA were found independent for serum PSA, Age and Gleason score whereas serum PSA levels correlated with Gleason score (all patients: *p *= 0.034; > 3 years follow-up: *p *= 0.024) but not with age. Interestingly SP Dkk-3 levels correlated with SP PSA levels (all patients: *p *= 0.013) but this correlation was limited to patients with biopsy confirmed PCa (all patients: *p *= 0.007; > 3 years follow-up: *p *= 0.026) while no correlation was observed in patients with negative biopsies (all patients: *p *= 0.301; > 3 years follow-up: *p *= 0.620).

**Table 4:** Correlative associations between univariate test variables.

		SP PSA			serum PSA			Age			Gleason*
					
		all	neg	pos	all	neg	pos	all	neg	pos	
		
Cohort	Variable	r (*p*-value)	r (*p*-value)	r (*p*-value)	r (*p*-value)	r (*p*-value)	r (*p*-value)	r (*p*-value)	r (*p*-value)	r (*p*-value)	r (*p*-value)
all patients	SP Dkk-3	-0.276	-0.165	-0.417	-0.950	-0.212	-0.128	0.118	-0.010	0.220	0.019
		(0.013)	(0.301)	(0.007)	(0.400)	(0.183)	(0.432)	(0.294)	(0.950)	(0.173)	(0.906)
	SP PSA	1	1	1	0.020	0.125	-0.068	-0.320	-0.107	0.037	0.114
					(0.860)	(0.437)	(0.679)	(0.777)	(0.507)	(0.822)	(0.483)
	serum PSA	0.020	0.125	-0.038	1	1	1	0.117	-0.107	0.024	0.336
		(0.860)	(0.437)	(0.679)				(0.298)	(0.507)	(0.884)	(0.034)
clinical follow-up	SP Dkk-3	-0.241	-0.104	-0.430	-0.100	-0.327	-0.174	0.080	-0.307	0.336	0.123
> 3 yr		(0.054)	(0.620)	(0.026)	(0.506)	(0.111)	(0.450)	(0.596)	(0.135)	(0.137)	(0.595)
	SP PSA	1	1	1	0.043	0.219	-0.099	0.099	0.071	0.169	0.181
					(0.777)	(0.293)	(0.668)	(0.511)	(0.844)	(0.465)	(0.431)
	serum PSA	-0.100	0.219	-0.099	1	1	1	0.085	0.312	0.018	0.491
		(0.506)	(0.293)	(0.668)				(0.572)	(0.129)	(0.938)	(0.024)

SP, seminal plasma; PSA, prostate-specific antigen; neg, negative biopsy; pos, positive biopsy; r, correlation coefficient.

Correlations were calculated by Pearson correlation analysis except for *Spearman's rank correlation analysis.

## Discussion

Serum PSA is currently the most widely used PCa screening test, however, PSA is organ- but not disease-specific, thus its value to predict PCa is limited and there is a need for additional disease markers. We consider SP as an ideal source to monitor pathological changes in the prostate since it more directly reflects local glandular production than other sources such as peripheral blood. Moreover, SP donation is completely non-invasive avoiding the discomfort and possible concerns associated with prostate massage necessary to obtain post-massage urine. Previously, we reported that the inactive free:total PSA ratios in SP from men with suspected PCa did not distinguish between those harboring malignant as opposed to benign disease [16]. The present study was designed to assess changes in Dkk-3 levels in SP and its potential for discriminating benign from malignant prostatic conditions in patients who were to undergo TRUS-guided biopsy due to elevated serum PSA (> 4 ng/mL) and/or abnormal DRE.

With the IEMA developed in our laboratory, SP Dkk-3 levels were found significantly higher in patients with biopsy confirmed PCa (100.9 ± 12.3 vs. 69.2 ± 9.4 fmol/mg). Of note, this difference was more pronounced in the subgroup of patients with a clinical follow-up of > 3 years (100.0 ± 16.6 vs. 56.3 ± 8.4 fmol/mg) for whom, as a consequence, there was greater veracity for the negative biopsy finding to be correct (Table 1).

While Dkk-3 levels as a single marker distinguished between prostate cancer and non-cancer on the basis of TRUS biopsies in the study cohort with a similar diagnostic accuracy compared with serum PSA (SP Dkk-3: AUC = 0.633; serum PSA: AUC = 0.644), the diagnostic accuracy of SP Dkk-3 levels was enhanced in multivariate models including serum PSA (model A) and both, serum and SP PSA levels (model B). Neither SP Dkk-3 nor SP PSA levels correlated with biopsy Gleason scores. However, this might be in part due to the patient cohort characteristics with approximately 2/3 of all positive biopsies reported as Gleason 7 (either Gleason 3+4 or Gleason 4+3). Of interest SP Dkk-3 negatively correlated with SP PSA levels in the positive biopsy cohorts, which might be responsible for the higher accuracy of model B compared with model A.

The biological significance of the increase in SP Dkk-3 levels in patients with positive vs. negative PCa biopsy findings remains unclear. As expression of Dkk-3 in secretory epithelial cells of the prostate is reduced in both PCa [5,13,20] and BPH [13], one might have expected a similar effect on SP Dkk-3 levels in patients with both these conditions. However, it is conceivable that the elevated Dkk-3 levels in PCa SP are derived from the tumor neovasculature, where high levels of Dkk-3 are produced [13]. Elevated serum PSA levels in PCa patients are supposed to be primarily an effect of tissue degradation and thus increased tissue permeability while in normal/BPH tissue an intact basement membrane prevents leakage [21]. Likewise, Dkk-3 as a secreted protein of similar size (PSA protein backbone: 237 aminoacids; Dkk-3 protein backbone: 329 aminoacids) might be able to diffuse through the disorganized PCa tissue.

Due to the limited number of SP samples available, especially from patients with long-term follow-up, it was not useful to further stratify the biopsy confirmed PCa patients according to their risk in order to more closely investigate the SP Dkk-3 and SP PSA correlation within this cohort.

Given the limited diagnostic accuracy of single markers including PSA, much effort has been made to identify potential additional PCa markers from different sources including SP [22-24]. Our data demonstrate that SP is a valuable and valid source for biomarkers of prostatic disease. The most promising attempt to improve PCa diagnosis appears the establishment of a set of individual biomarkers. Thus, SP Dkk-3 should be considered in a panel of markers such as prostate cancer antigen 3 (PCA3) and/or hepsin [25] to be evaluated in combination in future validation studies.

## Conclusions

A newly established sensitive and specific IEMA for Dkk-3 revealed significantly higher SP Dkk-3 levels in men with biopsy confirmed PCa indicating its inclusion in a PCa diagnostic marker panel. A multivariate model based on SP Dkk-3 and serum PSA levels showed higher diagnostic accuracy compared with the individual markers. Although mean SP PSA levels were not significantly altered in PCa patients, a combination of all three markers further improved results with the multivariate model, indicating potential utility in determining and integrating all three in a panel of biomarkers. However, more research is necessary to validate that SP Dkk-3 represents a clinically applicable PCa biomarker, ideally in a set of individual biomarkers.

## Competing interests

The authors declare that they have no competing interests.

## Authors' contributions

CZ performed Dkk-3 measurements and statistical analyses, participated in the design of the study and drafted the manuscript. MH carried out Dkk-3 and protein quantifications. HK performed PSA analyses. MB and RAG provided SP samples, RAG participated in the design of the study. HL performed mass spectrometry. PB conceived of the study, and participated in its design and coordination and helped to draft the manuscript. All authors read and approved the final manuscript.
